# Records of Daily Practice: A Scientific Visiting-List for Physicians and Surgeons

**Published:** 1860-01

**Authors:** 


*Records of Daily Practice: A Scientific Visiting-List for Physicians and Surgeons.*—-We have received from Ballière Brothers the above convenient little book, arranged by Dr. A. N. Bell, one of the Physicians to the Brooklyn City Hospital. It contains space for brief records of cases in daily practice, such as would be amply sufficient for unimportant cases, and serve as memoranda from which to write the fuller records of important diseases. The arrangement strikes us as exceedingly good, answering, at the same time, for a visiting-list and record-book. It is of a convenient size for the pocket, and will be exceedingly useful to physicians engaged in large practice. It can be obtained on application to the publishers.

